# Hibernation in an Antarctic Fish: On Ice for Winter

**DOI:** 10.1371/journal.pone.0001743

**Published:** 2008-03-05

**Authors:** Hamish A. Campbell, Keiron P. P. Fraser, Charles M. Bishop, Lloyd S. Peck, Stuart Egginton

**Affiliations:** 1 Department of Physiology, University of Birmingham, Edgbaston, Birmingham, England; 2 British Antarctic Survey, Natural Environment Research Council, Cambridge, England; 3 Department of Biological Sciences, University of Bangor, Bangor, Wales; University of Sheffield, United Kingdom

## Abstract

Active metabolic suppression in anticipation of winter conditions has been demonstrated in species of mammals, birds, reptiles and amphibians, but not fish. This is because the reduction in metabolic rate in fish is directly proportional to the decrease in water temperature and they appear to be incapable of further suppressing their metabolic rate independently of temperature. However, the Antarctic fish *(Notothenia coriiceps)* is unusual because it undergoes winter metabolic suppression irrespective of water temperature. We assessed the seasonal ecological strategy by monitoring swimming activity, growth, feeding and heart rate (*f*_H_) in *N. coriiceps* as they free-ranged within sub-zero waters. The metabolic rate of wild fish was extrapolated from *f*_H _recordings, from oxygen consumption calibrations established in the laboratory prior to fish release. Throughout the summer months *N. coriiceps* spent a considerable proportion of its time foraging, resulting in a growth rate (G_w_) of 0.18±0.2% day^−1^. In contrast, during winter much of the time was spent sedentary within a refuge and fish showed a net loss in G_w_ (−0.05±0.05% day^−1^). Whilst inactive during winter, *N. coriiceps* displayed a very low *f*_H_, reduced sensory and motor capabilities, and standard metabolic rate was one third lower than in summer. In a similar manner to other hibernating species, dormancy was interrupted with periodic arousals. These arousals, which lasted a few hours, occurred every 4–12 days. During arousal activity, *f*_H_ and metabolism increased to summer levels. This endogenous suppression and activation of metabolic processes, independent of body temperature, demonstrates that *N. coriiceps* were effectively ‘putting themselves on ice’ during winter months until food resources improved. This study demonstrates that at least some fish species can enter a dormant state similar to hibernation that is not temperature driven and presumably provides seasonal energetic benefits.

## Introduction

A number of temperate fish species become dormant during winter months [1]. During this time the fish remain inactive, cease feeding, and reduce protein synthesis and growth [2], [3]. However, dormancy in fish is thought to significantly differ from obligatory hibernating vertebrates [1], [2], [3], [4], [5], [6]. This is because the reduction in metabolism during winter correlates with the declining water temperature and can be overturned by temperature reversal [4], [5], [6]. Recent studies have found that otoliths from Antarctic Notothenioid fish display distinct growth annuli [7], [8], demonstrating that they too have seasonal variations in growth. The cessation of growth by Notothenioids during winter months appears paradoxical, because the Antarctic marine environment is considered one of the most thermally stable regimes on the planet [9] and these fish are often demersal omnivores living in shallow productive waters, where suitable prey are available all year round [10], [11], [12].

The Notothenioids include many species that remain in the inshore waters of the Antarctic continent and sub-Antarctic islands year round. This fish group has been overwhelmingly successful in the Southern Ocean and no other oceanic ecosystem is so dominated by a single taxonomic group of fish [13]. Previous studies that have tagged and recaptured Notothenioid fish, have demonstrated a 5-fold decline in growth rates from summer to winter months [12], [14], [15], [16]. It is very unlikely that the small seasonal change in near-shore Antarctic sea water temperatures is the major factor driving the observed large seasonal change in growth rates, as this would imply an unrealistic thermal sensitivity (a 5-fold change in growth for a seasonal 2°C temperature change). The virtual absence of solar radiation during winter, coupled with expansion of the sea ice, is responsible for producing amongst the lowest phytoplankton standing stocks anywhere on earth and, therefore, the lowest energy transfer through the marine food web [9]. Clarke [17] proposed that this temporal variation in food supply formed the basis of seasonal growth patterns in polar invertebrates. However, there is no evidence to suggest that the food supply of Antarctic fish is restricted during winter [10], [11], [12]. Moreover, the inshore fishes *Notothenia coriiceps* and *Harpagifer antarcticus* exhibit a reduction in feeding activity and a mobilisation of lipid reserves when acclimated to a winter photoperiod in the laboratory [12], [16], [18]. This decline in feeding occurred even when food was available in excess of that eaten; suggesting that decrease in growth rate during winter is a product of appetite suppression rather than food limitation *per se*. We hypothesised that the reduction in growth observed in Notothenioid fish during the Antarctic winter occurs due to a seasonal switch in ecological strategy, from one which maximises the procurement of food to another which minimises the energetic cost of living.

To test this hypothesis we have examined the behavioural and metabolic strategy of the Antarctic fish *Notothenia coriiceps,* a widespread omnivorous predator of the Antarctic and sub Antarctic inshore waters. Swimming activity, heart rate (*f*_H_) and metabolism were recorded by miniaturised electronic devices over a full annual cycle, as the fish responded to the annual physical and biotic fluxes of the Southern Ocean. From this extensive dataset, together with seasonal measurements of growth and feeding from wild fish, we show that *N. coriiceps* employs a hibernation–like ecological strategy during winter months. This finding is of profound interest, firstly because this type of ecological survival strategy has typically been considered to only occur in higher terrestrial vertebrates, and secondly, because these fish already live at the extreme lower thermal limit for physiological and metabolic processes.

## Results

### Growth and feeding

**Figure 1:**
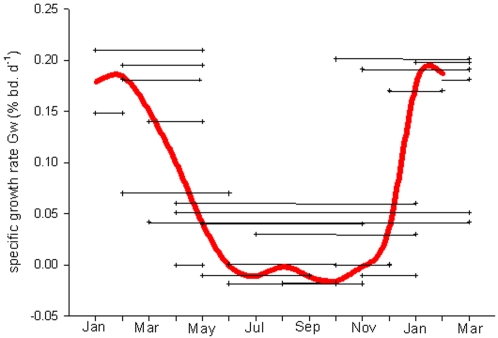
The mass specific growth rate (G_w_) of free-ranging and sea-caged *N. coriiceps.* A total of 21 immature adult *N. coriiceps* (4 recaptured twice) were tagged, released and then recaptured between 2^nd^ Jan 2004 and 12^th^ March 2005. The black crosses indicate the date of first and subsequent capture and the average G_w_ for an individual fish during its days at liberty indicated by the connecting black line. The red line connects calculations of G_w_ (n = 6) measured from sea-caged fish every 8 weeks. For clarity the standard error is not shown on the graph, which from May to Oct was <0.003% bd. wt. d^−1^, and from Nov to Apr <0.031% bd. wt. d^−1^).

A total of 118 immature adult *N. coriiceps* were caught by either fyke net or rod and line between Jan 2004 and Feb 2005. Of these, 21 were recaptured a second time within the year and 4 fish were recaptured more than once (Fig. 1). The highest specific growth rates (G_w_) were recorded in fish captured and recaptured between January–April (the austral summer) than at other times of year (Tukey HSD modified for unequal groups, F = 8.6, *P*<0.05, n = 25). The maximum G_w_ (0.21% bd. wt. d^−1^) was measured in a fish that was at liberty for 62 days between January and March. Nine fish that were tagged in the autumn and were recaptured in the early spring, thus the days of liberty only included winter months, exhibited close to zero or negative G_w_. Growth rates in these animals can be considered as representative of winter animals. The G_w_ of two fish that were at liberty for almost a full year was 0.052 and 0.041% bd. wt. d^−1^and are probably fairly representative of yearly field growth rates of *N. coriiceps* at Rothera.

It was necessary to keep the fish fitted with *f*_H_ dataloggers in sea-cages to enable serial sampling throughout the year. G_w_ was also determined in these fish on a bimonthly basis and showed a seasonal pattern similar to that seen in free-ranging fish (Fig. 1). That is, much higher growth rates in summer compared to winter. In February (summer) the G_w_ rate was 0.178% bd. wt. d^−1^. It declined rapidly after March (autumn) and reached a minimum of −0.02% bd. wt. d^−1^ in June (winter). During winter growth rates were negative, hence fish lost body mass. The decline in body mass continued until October (spring), after which the G_w_ rapidly increased, peaking at 0.172% bd. wt. d^−1^ in January 2004. The G_w_ from June to August was significantly lower than the G_w_ between December and February. (Tukey HSD, F_2, 10_ = 10.8, *P*<0.01). The within group variance was very low during winter months, when most fish showed negligible or negative growth rates, but had increased 10-fold by the summer when growth rate was high. This indicates that net energy gain differed between sea-caged fish; nevertheless, the large summer increase in G_w_ was still significant (ANOVA, F _2, 10_ = 19.1, *P*<0.01).

Capture rates during winter months were very low, but 5 fish were captured in July so allowing gut content analysis. These fish showed significantly (Tukey HSD test with modification for unequal *n* numbers F = 30.12, *P*<0.01, n = 5 & 9) less food in the gut (8.1±1.9 mg.g bd. wt.^−1^) than 9 fish captured in January (27.2±3.4 mg.g bd. wt.^−1^). The food in the gut of winter caught fish also consisted mainly of digested matter (61.42±5.4%), which composed a much smaller portion (18.17±3.2%) of the gut content of fish caught during summer (F = 24.3, *P*<0.01, n = 5 & 9).

### Activity

Fish movement was tracked continuously within a 1 km^2^ area using a static hydrophone array between March 2004 and March 2005. Only seven of the original 20 fish implanted with acoustic transmitters confined their complete daily and annual behavioural repertoire within the boundaries of the tracking zone. A sweep search every 100 m, up to 1 km outside the study area with a boat-mounted hydrophone, found 6 individuals 28–543 m outside of the study area. Other tagged individuals may have migrated further from the study site, but loss of study animals by transmitter fault or predation cannot be discounted. Even when fish were located within the tracking zone the software could not always determine location. This occurred because the acoustic pulse from the transmitter was not received by all 3 hydrophones in every instance and hence the position could not be triangulated. An in ability to fix individual fish occurred far more frequently during winter months. Investigation by SCUBA divers suggested fish that could not be fixed were located in crevices or under rocks, thereby blocking the transmitter signal.

**Figure 2:**
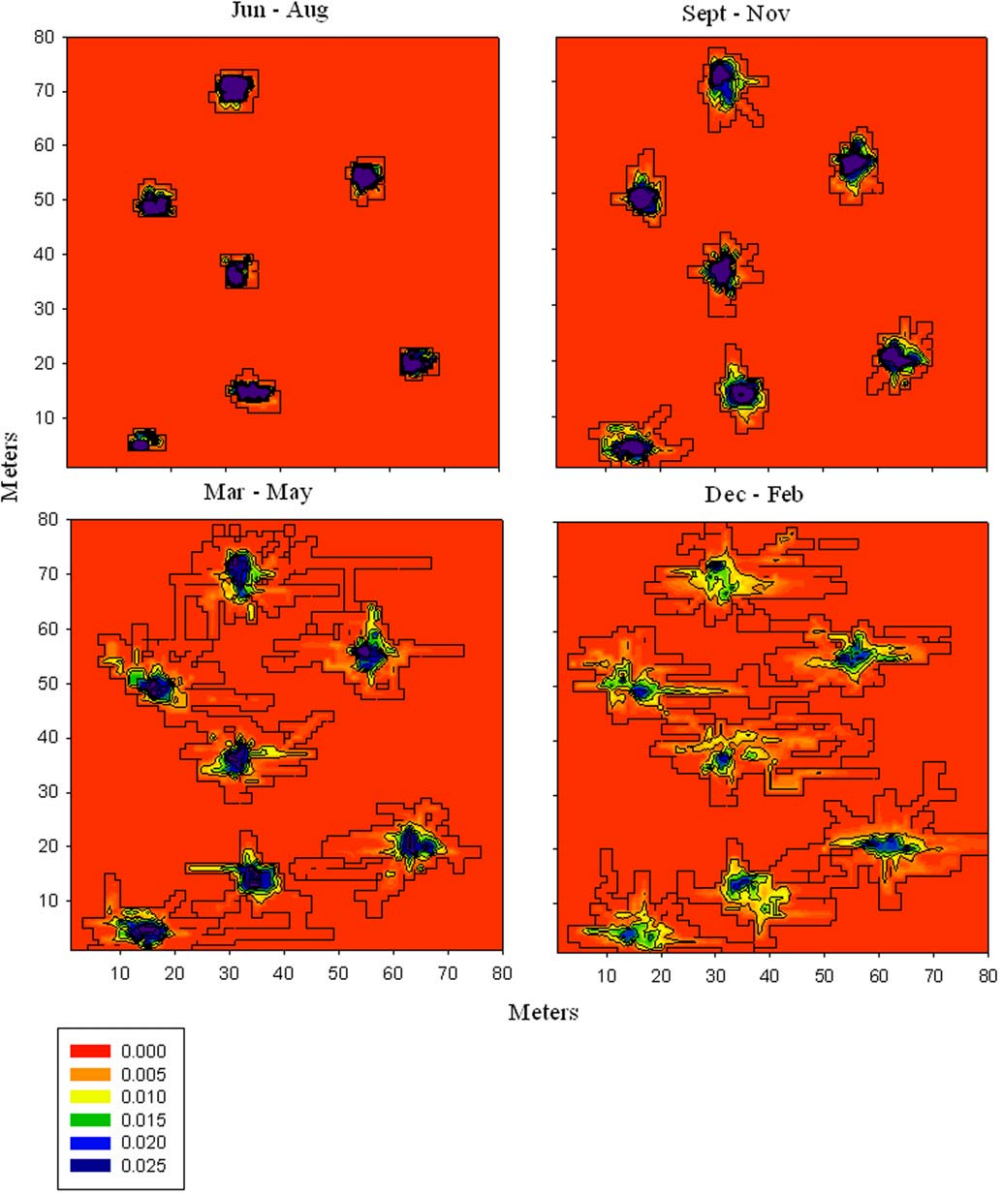
Seasonal home ranges of *N. coriiceps.* The precise locations of free-ranging fish were determined continually by acoustic telemetry throughout the year (n = 7). The 3-D spatial distribution plot was created by assigning positional data within 1 m^2^ cells over the range of the fish. The number of fixes per cell was calculated as a probability distribution of the total positional fixes for each 3 month period. The probability scale interval is 0.005, and purple areas indicate cells where the probability distribution was >0.03, the black outline indicates the outer boundary of the home range.

For each of the 4 designated seasons the fish showed a restricted home range, and movements within this area were non-random as tested by the Moran statistic (Table 1; *X* ^2^>0.25, *P<*0.05, n = 7). All fish showed high site fidelity to a central area of approximately 5–10 m^2^. However, the relative size of the home range, and the variance in probability distribution for the number of fixes made between cells, showed significant differences between seasons (Fig. 2, Table 1). Fish occupied a relatively large home range during summer months. However, ranges reduced in size during autumn and by winter occupied a 6-fold smaller area than during the summer. The mean daily swimming distance was reduced 20-fold between summer and winter, and the spatial distribution of *N. coriiceps* within its home range was concentrated within a much smaller core area during winter (Fig. 2, Table 1). In spring (Sept–Nov) the home range increased in size but the fish still concentrated a large portion of their activity within a small core area (5–10 m^2^). By December this had changed significantly (Table 1), and the fish were now spending equal proportions of their time at more locations throughout their home range. The maximum distance a fish traveled within an hour was also significantly greater (ANOVA, F _4, 5664_ = 54.05, *P*<0.01) in summer than in other seasons (Table 1).

**Table 1:** Activity parameters for free-ranging *N. coriiceps.*

	Mar–May *(autumn)*	Jun–Aug *(winter)*	Sep–Nov *(spring)*	Dec–Feb *(summer)*
**% total of position fixes**	31.2±12.2^a^	12.2±5.4^b^	23.4±8.8^c^	41.8±13.5^a^
**Size of range (m^2^)**	180.4±9.8^a^	60.8±3.4^b^	80.5±6.7^c^	233.0±14.2^d^
**Area spend >5% of time**	105±14.2^a^	27±2.4^b^	58±7.5^c^	137±18.4^a^
**Variance of Prob. Dist.**	4887±330^a^	18143±114^b^ 3	14453±90^c^ 1	4973±246^a^
**Total No tracking days (N = 7)**	546	598	532	560
**Days of no activity**	147	511	378	77
**Mean swimming speed (m h^−1^)**	12.1±3.2^a^	0.86±0.12^b^	5.3±2.3^c^	18.1±2.2^d^
**Max swimming speed (m h^−1^)**	24.3±5.2^a^	23.1±4.6^a^	21.3±3.1^a^	38.4±3.2^b^

Fish were tracked within a 1 km^2^ area by acoustic telemetry and a static hydrophone array over a 365 day period (mean±S.E, N = 7).

a,b,c,d indicates seasonal mean data that is significantly different as calculated by multivariate analysis (*P*<0.05).

### Heart rate and metabolism

**Figure 3:**
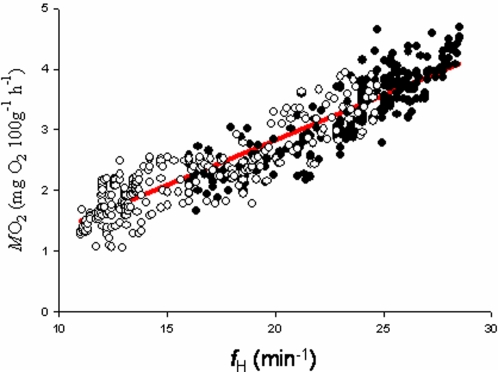
The seasonal relationship between heart rate (*f*_H_) and oxygen consumption (*M*O_2_) in *N. coriiceps.* Data were recorded in June (winter, open circles), and January (summer, closed circles). Fish were collected from sea-cages and immediately placed into a closed circuit respirometer, oxygen consumption and *f*_H_ were recorded simultaneously. ANOVA of fitted regression lines demonstrated that the slopes were not significantly different between summer and winter, and therefore a single regression line was fitted to explain the relationship between *f*_H_ and *M*O_2_ throughout the year (0.148 *X*–0.14, r^2^ = 0.86, N = 6 fish, n = 559 observations).

Prior to the fish fitted with *f*_H_ dataloggers being released into sea-cages, their *f*_H_ and oxygen consumption (*M*O_2_) were measured in the laboratory using a static respirometry chamber. There was a strong linear relationship (r^2^ = 0.86, n = 559) between *f*_H_ and *M*O_2_ for *N. coriiceps* (Fig. 3). Both *f*_H_ and *M*O_2_ showed similar ranges from minimum to maximum values in January and July. However, the whole range was displaced to lower values in both measures in winter. Therefore, the ratio of *M*O_2 _with *f*_H _did not change significantly between measurements taken in January and July (F = 2.54, P = 1.05, number of fish = 6, number of observations = 559), and a single overall regression equation (0.148 *X*–0.14, r^2^ = 0.86) was therefore used to estimate field metabolic rate from the *f*_H_ of sea-caged fish throughout the year.

**Figure 4:**
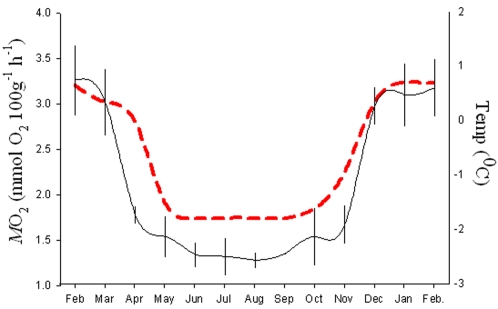
The free-ranging metabolic rate of *N. coriiceps.* The mean monthly *M*O_2_ of wild *N. coriiceps* (black line, n = 6) was extrapolated from continual field recordings of *f*_H_ using the equation given in Fig. 3. Water temperature was measured by an onboard temperature sensor (red).

The mean *f*_H_ recorded from fish inhabiting sea-cages during February was 25.2±1.2 min^−1^ and the estimated field *M*O_2_ for this month was 3.59±0.78 mg O_2_ 100 g^−1^ h^−1 ^(Fig. 4). Between February and April there was a 23% decline in *f*_H_ and therefore *M*O_2_. The fall in sea water temperature to the yearly low (−1.8±0.02°C) occurred in mid-April, and thus succeeded the decline in *N. coriiceps* metabolism. Between June and October *f*_H_ showed little variability and remained around 11±0.8 min^−1^ with a calculated metabolic rate of 1.48 mg O_2_ 100 g^−1^ h^−1^. Consequently, summer and winter metabolism differed by 58%. During November and December *f*_H_ and water temperature steadily increased, however by mid-December water temperatures reached the summer maximum of 0.7±0.02°C whilst *f*_H_ and *M*O_2_ continued to rise through December until February.

In a separate, controlled laboratory experiment we measured standard metabolic rate (SMR) and resting *f*_H_ in starved non-swimming *N. coriiceps.* The difference between summer and winter standard metabolism in these fish was 29% (T _2, 12_ = 3.6, P<0.01, ). Reversing the small difference between summer and winter water temperatures produced no significant change (T _2, 12_ = −0.4, P = 0.64) in *f*_H_ or *M*O_2_ of laboratory fish (Table 2).

**Table 2:** The effect of altering winter and summer water temperature on *f*_H_ and *M*O_2_ in *N. coriiceps*.

	January		June	
	*M*O_2_ 100 g fish (mg O_2_ h^−1^)	*f*_H_ (min^−1^)	*M*O_2_ 100 g fish (mg O_2_ h^−1^)	*f*_H_ (min^−1^)
**Ambient temperature**	2.72±0.21^a^	19.3±2.4^b^	1.95±0.20^c^	14.1±2.6^d^
**Switched seasonal temp**	2.64±0.25^a^	18.8±2.1^b^	2.05±0.24^c^	14.8±2.1^d^

a,b,c,d are used to denote significantly different means as tested between rows (Students paired t-test, *P*<0.05).

### Periodic arousals

**Figure 5:**
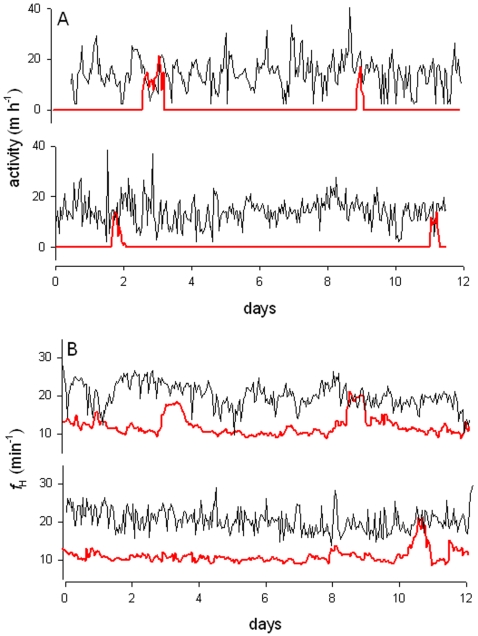
Summer and winter comparison of hourly activity and *f*_H_ profiles in *N. coriiceps.* Panel A shows activity profiles from two separate fish remotely recorded by acoustic telemetry during summer months (black), and the activity profile for the same fish is also shown during winter months (red). The flat line indicates when the fish were sedentary. Panel B shows the mean hourly *f*_H_ from two sea-caged *N. coriiceps* during summer (black) and *f*_H_ for the same fish during winter (red).

The activity of free-ranging fish during summer months varied between 0 and 38.4 m h^−1^, with no evident circadian rhythm. This contrasted to winter months when the fish were sedentary for much of the time, but exhibited short bouts (1–3 hours) of activity where rates were similar to summer. These short bouts of activity in winter occurred between every 4 to 12 days (Fig. 5A).

A similar seasonal profile was seen in the *f*_H_ of fish held in sea-cages. In summer *f*_H_ was very variable and ranged between 12 and 26 beats min^−1^, and there was no evidence of circadian rhythmicity (Fig. 5B). During winter months the *f*_H_ was less variable and >2-fold slower than during the summer months (ANOVA, F _2, 55447_ = 584, *P*<0.01).The low winter *f*_H_ was intermittently elevated every 4–12 days for periods lasting a few hours. During these periods *f*_H_ was elevated to rates similar to summer months.

**Figure 6:**
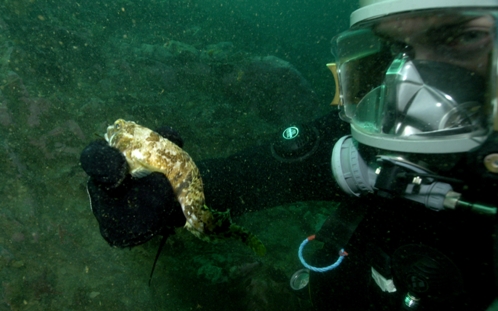
SCUBA diver holding a non-responsive hibernating *N. coriiceps.* The fish was collected from under the Antarctic sea-ice in August from 18 m depth, temperature −1.8°C.

SCUBA divers visiting winter refuges of fish in which movement could not be detected by acoustic telemetry, found the fish to be initially unresponsive to handling (Fig. 6). After 20 to 60 sec of handling the fish would become active and swim off, albeit sluggishly. In summer SCUBA divers could not handle wild *N. coriiceps* in this way.

## Discussion

The Antarctic marine environment is characterised by continual near-freezing temperatures, and highly seasonal primary productivity driven by the lack of light in winter [17]. In these conditions the Antarctic Notothenioid fish have flourished, although the ecological strategy adopted by these fish to endure the Antarctic winter is poorly understood. Data reported here from field observations over an annual cycle illustrate, for the first time, a tactic of metabolic suppression that displays many parallels with hibernation-like responses from higher vertebrate classes.

*Notothenia coriiceps* in this study were exposed to natural conditions typical of the Southern Ocean: large seasonal differences in photoperiod and primary productivity, but little variation in temperature. Free-ranging and sea-caged *N. coriiceps* in our study exhibited a significant winter decrease in activity, *f*_H_, metabolism and growth. Previous studies on Antarctic notothenioids have also demonstrated a winter reduction in growth rates [10], [11], [12], [14], [15], [16]. *N. coriiceps* is likely to have exhibited a loss of mass during winter due to reduced feeding and a reliance on endogenous lipid reserves [18]. Prey capture is likely to be associated with foraging activity. There could be many reasons for the reduction in foraging activity, including a reduced ability in a visual predator to capture food in low light conditions in winter. Irrespective of the underlying cause, the sedentary behaviour of the fish during winter months partially explains the reduction in weight. This winter switch in ecological strategy, from one which capitalizes on energy gain through foraging to another which curtails the energetic cost of metabolism, is a common theme for hibernating organisms [19].

The current study estimated *M*O_2_ from field recordings of *f*_H_, and for the first time determined field metabolism in a fish from polar waters. This was possible because cardiac output (CO) is a robust indicator of metabolic rate [20] and Antarctic fish modulate CO chiefly by changes in *f*_H_ [21]. In *N. coriicep*s the oxygen pulse (*M*O_2_: *f*_H_) did not vary between summer and winter seasons, and therefore, confirmed that for this species *f*_H_ was a highly reliable indicator of metabolism. Our results demonstrated a 58% suppression in the total metabolic rate (TMR) from wild *N. coriiceps* between summer to winter months, and the seasonal difference in water temperature was not responsible for the seasonal shift in *f*_H_ or *M*O_2_. The active suppression of *M*O_2_ and *f*_H_, irrespective of temperature, is a novel observation in fish and may correspond to the metabolic rate depression at the onset of hibernation of other vertebrates, where entry is also independent of body temperature [22].

The disparity in standard metabolic rate between the winter dormant period and the summer active period was relatively small (29%) compared to that of hibernating endotherms [22]. Nevertheless, the difference between summer and winter metabolism occurred in the absence of thermoregulatory requirements, and suggests an active suppression of physiological processes. Indeed, *f*_H_ was reduced 2-fold and food processing may have been down-regulated in dormant *N. coriiceps* (as implied by the 3-fold increase in digested matter found in the gut of wintering compared to summer fish). Laboratory studies on seasonally acclimated *N. coriiceps* have also reported a winter depression in muscle and liver enzyme activity [23], and the non-responsiveness of *N. coriiceps* to human handling when taken from a winter refuge was reflective of the reduced sensory and motor capabilities documented in hibernating species, another energy saving physiological response [25].

In this study, although a significant seasonal difference was found in the metabolic rates of laboratory held fish, the change from summer to winter standard metabolic rates was much less than that recorded in wild *N. coriiceps*. A possible reason for the lack of dormancy behaviour in the laboratory fish may be due to a disturbance effect, which is well documented to interrupt hibernation and delay the entry into dormancy of many hibernating animals [23]. This may also explain why previous studies that transported *N. coriiceps* outside Antarctica, where little or no seasonal signal was evident, and then acclimated those fish to seasonal conditions in the laboratory observed negligible metabolic suppression [18], [24].

The suppression of standard metabolism and *f*_H_ during winter correlated with the cessation of activity. Interestingly, wintering *N. coriiceps* showed short periodic episodes when both *f*_H_ and behavioural activity were elevated to summer rates. This demonstrated that during the dormant period *N. coriiceps* maintained the ability to up- or down-regulate metabolic and physiological processes and undertake swimming activity. The reason for such arousals is at present unclear, but the necessity of *N. coriiceps* to partake in these energetically expensive arousals draws further parallels with other hibernating groups. Why hibernating animals undergo expensive arousals is a subject of much speculation, and along with the proximal signals remain a long-standing, unresolved question of hibernate research [25].

It is considered that the ‘Zeitgeber’ for most animals to enter seasonal hibernation is either environmental temperature or photoperiod [4], [5]. Fish have an overt sensitivity to light and the seasonal extremes of photoperiod at high latitudes in the absence of thermal change make it an obvious environmental cue for *N. coriiceps*. Polar fish certainly have the ability to anticipate winter conditions, e.g. Atlantic wolf fish held in constant darkness upregulate antifreeze proteins and blood electrolytes to protect themselves from expected winter ice crystal formation [26]. Most hibernators seek constant darkness to assist with entry into hibernation and this has recently been shown to stimulate the expression of genes facilitating hibernation in mammals [27].

### Conclusions

Hibernation is a complex subject, with animals continually being described as hibernators that challenge traditional views [28], [29].The winter dormancy we have documented in the Antarctic *Notothenia coriiceps* is distinct from the facultative dormancy observed in temperate fish species by the levels, and duration of the reduced physiological state, and is instead similar in many ways to the state achieved by truly hibernating species. There may be a continuum between mild levels of metabolic depression seen in many species over short timescales to full hibernation where dormancy in *N. coriiceps* forms a significant link. The seasonal hibernation strategy observed in *N. coriiceps* may be common to many, if not all Notothenioid fishes as illustrated by otolith growth annuli [7], [8]. This, and the ability to anticipate and compensate for marked seasonal effects may have contributed to the success of the Notothenioids in the Southern Ocean. Finally, the ability of *N. coriiceps* to actively suppress physiological processes beyond what is generally viewed as the extreme lower thermal threshold highlights that the ‘over-wintering’ Antarctic fish may be harbouring further cellular secrets.

## Methods

### Study site and fish

The study was conducted in a 1 km^2^ inshore area off Rothera Research Station (British Antarctic Survey), Adelaide Island (67° 34′S 68° 08′W), Antarctica. *Notothenia coriiceps* (457±28 g, n = 166) were caught by fyke net and only immature adults were selected for the study, in order to avoid factors associated with reproductive cycles. Seasonal variation in growth has been shown to have no gender dependence and therefore this was not taken into consideration when sampling [10], [14]. After capture fish were taken immediately to an aquarium at ambient sea water temperature and photoperiod. All surgical procedures for tag attachment were undertaken within an air-cooled room (0°C), and the fish were anaesthetised in MS222 (0.3 g l^−1^), before being placed on an operating table where their gills were irrigated with aerated seawater containing MS222 (0.1 g l^−1^).

### Growth and feeding of free-ranging fish

Between Jan 2004–Mar 2005 118 *N. coriiceps* (418±13 g) were caught, weighed, their lengths determined, and a numbered T-bar anchor tag (FD-64, Floy tag, Seattle, U.S.A.) fitted between the dorsal rays. The procedure took less than 2 min and the fish were returned at point of capture. Fish which were subsequently recaptured were measured, weighed and released, following weekly net-laying. Specific growth rate (G_w_ in % body weight day^−1^) was calculated using the following equation [14]:
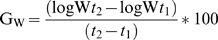
Where: W = body weight 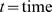



Gut content analysis was undertaken on 5 *N. coriiceps* (425±28 g) captured by fyke nets in winter months and 9 (489±22 g) captured during summer. The fish were weighed and total gut content expressed per mg of total fresh body mass.

### Acoustic tracking

In March 2004, 20 *N. coriiceps* (436±28 g), were implanted with an acoustic transmitter (VS8, Vemco Ltd, Nova Scotia, Canada), which emitted a pulse every 15–30 s and had an acoustic range of 250m. The transmitter, which had a battery life of 400 d, was surgically implanted into the peritoneal cavity *via* a 2 cm incision made behind the left pectoral fin. The muscle was closed using an interrupted stitch with a 5/0 catgut suture and the skin closed using a non-interrupted stitch with 5/0 nylon monofilament; surgery took <12 min. The fish were recovered and kept under observation for 24 h before being released at the point of capture. Fish position was determined in the field using a purpose built static hydrophone array, consisting of three fixed acoustic receivers (VR2 receiver, Vemco Ltd) with a detection radius of 210 m. These were anchored to the seabed using climbing pitons. Receivers were placed 130 m apart and floated 2–9 m above the sea-bed, so that each was at the same depth (depth was accounted for when fish position was determined). The exact location in longitude and latitude coordinates of each hydrophone was determined by a hand held GPS system (GPS 76, Garmin, U.S.A.), taking the average latitude and longitude measurements over a 5 min period at the water surface directly above the hydrophone. ARCGIS software used these coordinates to plot position onto a scaled, orthorectified aerial photograph of the study area (MAGIC, British Antarctic Survey). The position of each fish that carried an acoustic transmitter was calculated by triangulation based on the relative position of each hydrophone, the speed of sound through the water and the difference in timing of the pulse arriving at each specific hydrophone. Pilot studies found this method to be accurate to <0.3 m.

### Recording heart rate from fish held in sea-cages

In February 2004, miniature electronic micro-controlled data loggers (DL), capable of making high resolution recordings of ECG during free-ranging activity were attached to 6 *N. coriiceps* (589±34 g). The DL and housing was neutrally buoyant in water. A rubber saddle was permanently secured through the dorsal rays of the fish with nylon T-bar tags (FD-64, Floy tag Seattle, U.S.A), and the ECG electrodes (0.2 mm, Teflon coated 7-strand stainless steel wire, A–M systems, Connecticut, U.S.A.) were placed subcutaneously using a hypodermic needle a few mm through the septum behind the 4^th^ left gill arch. The saddle and recording electrodes could be quickly attached or detached from the DL. The ECG was analysed *in situ* by a microprocessor using proprietary software that performed waveform analysis to generate inter-beat intervals and, thus, instantaneous *f*_H_. The logger was primarily used in inter-beat mode but was also programmed to record two complete ECG waves every 4000 beats, to validate the quality of the ECG signal and the veracity of the calculated *f*_H_ (*details in* 30). The DL also recorded ambient temperature every minute to a resolution of ±0.3°C. Fish fitted with the DL were housed individually within 4 m^3^ cages secured to the sea bed.

The DL needed to be exchanged every 60 d for battery replacement and data download. To enable DL exchange with minimal disturbance to the fish a cylindrical tube 20 cm D×50 cm L with double threaded end caps was placed in each pen, in which the fish would naturally seek refuge. The fish would always retreat inside the refuge when a SCUBA diver was in close proximity. The diver would open the pen, attach threaded end caps to both ends of the refuge, and remove it from the pen. On arrival at the water surface the refuge was immediately submerged into a large insulated holding tank containing sea water at ambient temperature then transported to an air temperature controlled aquarium approximately 100 m away. In the aquarium, each refuge was submerged in light anaesthetic (MS222 0.1 g^−1^) for 5 min. Once anaesthetized the fish was removed and placed on an operating table where its gills where flushed with fresh aerated sea water, to commence recovery. The housing cap was removed and the datalogger quickly exchanged (<2 min). The caps were secured onto the refuge and submerged in a large holding tank. The fish was returned to its original sea cage using the reverse of the collection procedure.

The cages had a large mesh size so that smaller prey items could enter the sea-cage, but to assist in attracting small invertebrate prey items a bait ball of chopped fish was inserted in the cage every month.

### Extrapolation of field *M*O_2 _from *f*_H_

Prior to release into the sea-cages, in January and June 2004 fish were first placed into a cylindrical respirometry chamber (8 cm D×30 cm L; vol 5 l) for 72 hours. All chambers were immersed into an aerated water bath that was continually flushed with fresh sea water at ambient sea temperature. Each chamber was fitted with two submersible pumps (100 l h^−1^, Interpet, UK), one circulated the water around the chamber whilst the other flushed the chamber with aerated water from the water bath. This created a flow rate of 0.3 cm s^−1^ within the chamber. Water was extracted automatically from each chamber in series, *via* a rotor valve (Omnifit, Birmingham, UK), and injected into a purpose made flow cell containing a polarographic oxygen electrode (Strathkelvin, Glasgow, UK). The oxygen concentration from each chamber was sampled at 1 Hz for 15 minutes every two hours and during this period *f*_H_ was recorded simultaneously by the attached data logger (see above). The relative oxygen depletion was calculated as a rate constant (only depletion traces with an r^2^>0.90 were used in calculations). Adjustments were made for the dissolved oxygen in seawater at ambient temperature, the volume of the chamber, and the mass of the fish and subsequent water displacement. *M*O_2_ values were then converted by the mass exponent for summer (0.82) and winter (0.76) immature adult *N. coriiceps* [22].

In a separate respirometry study, *N. coriiceps* (343±23g, n = 6) caught by fyke net in January and July were held in a large circular tank held at ambient sea temperature under local photoperiod. The instantaneous ECG and *M*O_2_ were recorded in fish after 72 h undisturbed rest in a respirometry chamber (as above). After the recordings were made on resting fish the seasonal temperature was reversed so that the June external water bath (−1.8°C) was warmed to 1°C, and in January lowered to −1.8°C. Recordings of *f*_H_ and *M*O_2_ were made 48 h later. Photoperiod was kept as per local conditions i.e. constant light in January and darkness in June.

### Statistical analysis

Variance in activity parameters was tested using the F-test statistic and the means compared by multivariate analysis. To test that fish movement within the home range was not random, a weighting or connectivity matrix was generated to represent the spatial arrangement of each cell. The Moran statistic was employed to determine if data values within a cell were influenced by data values of other nearby cells [31]. Seasonality effects or the effects of treatment on mean physiological parameters were tested using the Students-paired t-test or Durban Watson statistic for serial autocorrelation. To test for seasonal shifts in specific growth rate the Tukey HSD test was employed and fish were grouped depending on the season which the initial and subsequent measurements were recorded. Sometimes this statistic needed to be adjusted for unequal size of groups. All statistics were applied using Statgraphics 5.1 or Minitab 12.0 software and were deemed significant at P<0.05.
